# Correlations between the semiologic changes and the imaging aspects in the lateral bulbar infarction

**Published:** 2016

**Authors:** I Cristea, C Popa

**Affiliations:** * Neurologic Clinic, National Institute of Neurology and Cerebrovascular Diseases, Bucharest, Romania; “Carol Davila” University of Medicine and Pharmacy, Bucharest, Romania; ** ”Carol Davila” University of Medicine and Pharmacy, Bucharest, Romania; Romanian Society of Stroke; Neurology Department, National Institute of Neurology and Cerebrovascular Diseases, Bucharest, Romania

**Keywords:** lateral medullar infarction, intra-cranial vertebral artery, posterior inferior cerebellar artery, 3D TOF MIP

## Abstract

The study aimed to evaluate the correlations between the clinical and paraclinical data in the lateral bulbar infarction, benefiting from the access to the semiologic characteristics of a group studied and the MRI angiography, without a contrast agent, through the 3D TOF technique combined with MIP, as an imaging technique for the evaluation of the arterial lesion. The study group included 20 patients with lateral bulbar infarction, 14 men, and 6 women aged between 21 and 80 years, the mean age being 56, 9 years, who were enrolled in the study in the period 2012 and 2014, following the admission in the National Institute of Neurology and Neurovascular Diseases. All the patients enrolled in this stage study, performed brain MRI - in the Medinst laboratory, which included the following sequences T1, T2, Flair, DWI, MRI angiography without contrast agent (3D TOF combined with MIP). The study was retrospective. Following the analysis of the 3D TOF sequences combined with MIP, it was found that in the group studied, 8 patients had damage at the level of the vertebral artery, 2 at the level of the posterior inferior cerebellar artery and 10 patients presented mixed lesions of both the vertebral artery and of the PICA artery. In terms of the mechanism involved, most of the lateral bulbar infarctions were generated by arterial dissection (9 cases) and 6 cases had atheroma as etiology. Regarding the risk factors, dyslipidemia and smoking predominated in the studied group and the most common signs and symptoms were gait abnormalities, the ataxia of the limbs, dysphonia, and Horner syndrome.

**Abbreviations:** 3D TOF = 3D time of flight angiography, MIP = maximum intensity projection, MRI = magnetic resonance imaging, CT = computed tomography, FLAIR = fluid attenuated inversion recovery, DWI = diffusion weighted imaging, HTA = hypertension, DZ II = diabetes mellitus, VA = vertebral artery, PICA = posterior inferior cerebellar artery, VG = vertigo, NT = nystagmus, N/ E = nausea/ emesis, DP = dysphagia, PVP = pharyngeal/ vocal cord paresis, HS = Horner syndrome, PTH = pain/ temperature hypesthesia, LA = ipsilateral limb ataxia, GA = Gait ataxia, C-R-F = Cardiovascular risk factors, L = left, R = right

## Introduction

The lateral bulbar infarction is easily recognizable due to the appropriate signs and symptoms, which fall within the Wallenberg syndrome. It is responsible for about 2% of all the strokes [**1**]. The diagnosis of this syndrome often depends on the clinical symptoms and signs. The nuclear magnetic resonance has become the preferred imaging method in studying the lateral bulbar infarction due to the spatial resolution and the increased contrast between the normal and pathological tissues [**2**]. Some of the vascular causes responsible for the lateral bulbar infarction are the damage of the vertebral artery or of the posterior inferior cerebellar artery by dissection, thromboembolism, or atherosclerosis. The angiography with a digital subtraction is the gold standard for the diagnosis of the arterial lesions at the level of the vertebral artery, and of the posterior inferior cerebellar artery with a CT angiography and MRI angiography. However, the angiography with a digital subtraction is an unsuitable investigation for the screening of the arterial lesions due to the invasiveness and risk of an iatrogenic vascular accident [**3**]. The advanced MRI techniques are available for the detection and assessment of the atherosclerotic plaques and of the vascular wall at the level of the intracranial arteries [**3**,**4**]. The study aimed to evaluate the correlations between the clinical and paraclinical data in the lateral bulbar infarction, having as a technique for the assessment/ description of the arterial lesion, mainly the MRI angiography, without contrast agent, through the 3D TOF technique combined with MPI.

## Materials and methods

20 patients with lateral bulbar infarction were included in the study and were enrolled between 2012 and 2014, out of whom 14 were men and 6 were women, aged between 21 and 80 years, the mean age being 56,9 years. The group of patients hospitalized with lateral bulbar infarction was much larger in the time period mentioned above but the patients to whom brain MRI was not performed with 3D TOF sequence combined with MIP, but only brain CT were excluded together with the patients who died within the first 24 hours from the admission. The study was retrospective, including patients admitted in the National Institute of Neurology and Neurovascular Diseases. All the patients enrolled in this stage study, performed a brain MRI - in the Medinst laboratory, which included the following sequences T1, T2, Flair, DWI, MRI angiography without a contrast agent (3D TOF combined with MIP). Two of the investigations had an MRI angiography with a contrast agent to confirm the lateral bulbar infarction and to discover the etiology of the artery involved.

The MRI images were performed on a 1, 5 T scanner (Magnetom Avanto A Tim plus Dot System, Siemens). TR/ TE 25/ 7, SL between 0, 69-1 FOV (field of view) 180x180, matrix 241x256 was used in the 3D TOF transverse sequence plus MIP (used to describe the pathology of the vessel).

The vascular lesions at the level of the vertebral artery in the lateral bulbar infarctions were classified into atheromatous lesions, arterial dissection, thromboembolism, and no arterial damage.

The arterial dissection is thus defined in the 3D TOF sequence as true and false double lumen at the lesion place, the atheroma is defined as the thickening of the wall between the lumen and the vessel wall, and the thromboembolism is defined as a damage of the arterial wall that is not fulfilling either the criteria of dissection or of atheroma [**3**,**4**]. 

## Results

There were 14 men and 6 women, aged from 21 to 80 years (mean age 56, 9). The risk factors and neurological symptoms/ signs are summarized in **Table 1**. The lateral bulbar infarction occurred more frequently in men from all the patients in the study group.

**Table 1:** Clinical, MR findings and risk factors in 20 patients with lateral medullary infarction

Case	Age(y)/ Sex	Signs & symptoms	Stroke risk factors	Lesion localization of LMI
1	37/ F	VG,N/ E, PVP,PTH, LA, GA, HS	without risk factors	left
2	44/ F	PVP, DP, HS,PTH, LA, GA	smoking, hyperlipidemia	left
3	57/ B	VG, NT,DP, PVP, HS, LA, PTH, GA	Smoking, hypertension	right
4	59/ F	VG, NT,PVP, DP, HS, PTH, LA, GA	hypertension, hyperlipidemia	right
5	80/ B	NT, PVP, HS, PTH, LA, GA	hypertension, hyperlipidemia	left
6	77/ B	DP, PVP	smoking, hyperlipidemia	right
7	21/ B	N/ E, NT, LA, PTH, GA	without risk factors	right
8	52/ B	VG, N/ E, GA	without risk factors	left
9	67/ F	VG, N/ E,NT, DP, PVP, HS, PTH, LA, GA	atrial fibrillation	right
10	70/ B	DP, PVP, PTH, LA, GA	Smoking, diabetes mellitus	right
11	66/ B	LA, GA	hypertension, diabetes mellitus, hyperlipidemia	right
12	40/ B	DP, PVP, HS, PTH, LA, GA	without risk factors	left
13	55/ B	VG,N/E, PVP, PTH, LA, GA, HS	without risk factors	left
14	57/ F	PVP, DP, HS, PTH, LA, GA	smoking, hyperlipidemia	right
15	78/ B	VG, NT,DP, PVP, HS, LA, PTH, GA	hypertension, hyperlipidemia	right
16	75/ B	VG, NT, PVP, DP, HS, PTH, LA, GA	hypertension, hyperlipidemia	left
17	20/ B	NT,DP, HS, PTH, LA, GA	hypertension, hyperlipidemia	right
18	50/ B	DP, PVP	smoking, hyperlipidemia	right
19	65/ F	N/ E, NT, LA, PTH, GA	without risk factors	left
20	68/ B	VG, N/ E, GA	without risk factors	right

Correlations between the typical clinical manifestations that occur in the lateral bulbar infarction and the anatomical structures involved are presented in **Table 2** [**5**]. 

**Table 2:** Signs, symptoms, and structures involved in lateral medullary infarction

STRUCTURES INVOLVED	SIGNS AND SYMPTOMS
Descending tract and nucleus of the fifth nerve	Pain, numbness, impaired sensation over half the face
Vestibular nuclei and connection	Vertigo, nausea, vomiting, nystagmus, diplopia, oscillopsia
Descending sympathetic tract	Horner syndrome (miosis, ptosis, decreased sweating)
Issuing fibers ninth and tenth nerves	Dysphagia, hoarseness, paralysis of vocal cord, diminished gag reflex
Restiform body, cerebellar hemisphere, olivocerebellar fibers, spinocerebellar tract	Ataxia of limbs, falling to side of lesion
Cuneate and gracile nuclei	Numbness of ipsilateral arm, trunk or leg
Spinothalamic tract	Impaired pain and thermal sense over half the body, sometimes face

Thus, the pain, the numbness and the objective sensitivity disorder at the level of the ipsilateral hemiface are given by the damage of the descending tract and of the V nerve nucleus, the vertigo, nausea, vomiting, nystagmus and diplopia occurring while affecting the vestibular nuclei, the ipsilateral Horner syndrome, by damaging the descending sympathetic tract; dysphagia, dysphonia, the ipsilateral vocal cord paresis by affecting the IX and X nerve fibers, the ataxia of the ipsilateral limbs, the fall in the side of the lesion, and the sensation of lateropulsion occur through the damage of the restiform body, of the olivocerebellar fibers, of the cerebellar hemispheres, and of the spinocerebellar tract. Paraesthesia at the level of the ipsilateral arm, torso, and leg may also occur in the Wallenberg syndrome by affecting the cuneate and gracilis nucleus and the spinothalamic tract. A damage of the contralateral thermoalgic sensitivity of the torso and limbs and, more rarely, ipsilateral, may also occur.

**Fig. 1:**
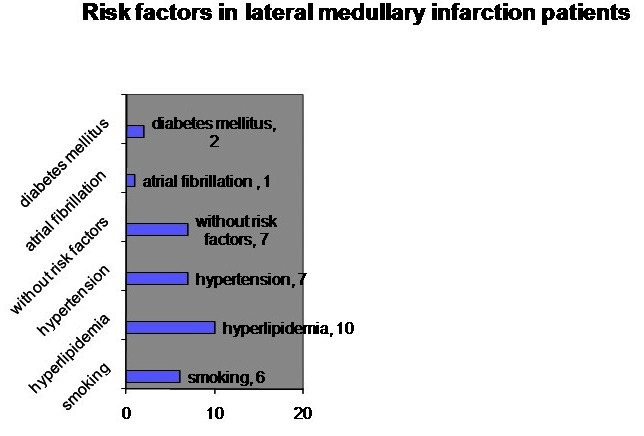
Risk factors in lateral medullary infarction patients

Regarding the risk factors of the patients included in the study, 7 patients had HTA, 2 patients had diabetes mellitus, 6 were smokers, 10 had hyperlipidemia, 1 patient presented cardiovascular risk factors and 7 patients did not present risk factors (**Fig. 1**).

**Fig. 2:**
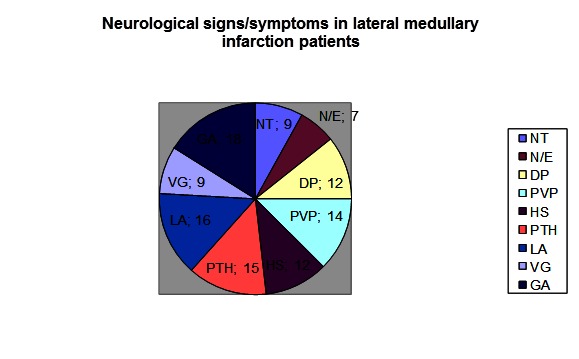
Neurological signs/ symptoms in lateral medullary infarction patients

In terms of symptomatology, out of the 20 patients, 9 presented vertigo, 9 nystagmus, 16 ipsilateral limb ataxia, 18 gait ataxia, 7 nausea/ emesis, 12 dysphagia, 14 pharyngeal/ vocal cord paresis, 12 ipsilateral Horner syndrome and 15 pain/ temperature hypesthesia (including the objective sensitivity disorder at the level of the ipsilateral hemiface, paraesthesia at the level of the ipsilateral arm, torso and leg, the damage of the contralateral thermoalgic sensitivity of the torso and limbs, and, more rarely ipsilateral damage). 8 lateral bulbar lesions were on the left and 12 on the right **Fig. 2**.

In the study group, out of the 20 patients included in the study, 8 presented lesions located strictly at the level of the vertebral artery, 10 had mixed damage of VA and PICA, two patients presented PICA damage, without lesions at the level of the vertebral artery (**Table 3**).

**Table 3:** Detection of Arterial lesions in 20 patients with lateral medullary infarction

Vertebral artery only	8
VA + PICA	10
PICA only	2

According to the criteria above, after analyzing the imaging investigations (the examination of the 3D TOF sequences combined with MIP), a classification of the type of lesion present at the level of the vertebral artery (to which also the cervical-cerebral Doppler ultrasonography performed in patients during hospitalization were taken into account in addition to the imaging changes) was presented: out of the 20 patients included in the study, 9 patients presented an arterial dissection - according to the criteria mentioned above, 6 had the atheroma as vascular etiology, and one patient did not meet either the criterion of dissection or of atheroma (thromboembolism) (**Table 4**).

**Table 4:** Arterial lesion in Vertebral Artery on 3D TOF angioMRI with MIP

	ATHEROMA	DISSECTION	THROMBOEMBOLISM	NONE	TOTAL
STENOSIS	3	5	-	-	8
OCCLUSION	3	4	1	-	8
NONE	-	-	-	4	4
TOTAL	6	9	1	4	20

A further study of the characteristics of the group of patients included in the study from the group of patients with PICA plus damage (meaning the involvement of both the vertebral artery and the posterior inferior cerebellar artery), 8 of them were men out of whom: 3 presented vertigo, 3 nystagmus, 5 nausea/ emesis, 4 dysphagia, 4 vocal cord paresis, 1 Horner syndrome, 5 pain/ temperature hypesthesia, 5 ipsilateral limb ataxia and 7 gait ataxia. In the other group, which involved only the vertebral artery, 5 patients were men, 4 presented vertigo, 3 had nystagmus, 3 had nausea/ emesis, 4 presented dysphagia, 7 presented pharyngeal/ vocal cord paresis, 8 had Horner syndrome, 8 had pain/ temperature hypesthesia, 8 had ipsilateral limb ataxia, 8 had gait ataxia (**Table 5**).

**Table 5:** The comparison of neurological signs/ symptoms between the groups with or without a Posterior Inferior Cerebellar Artery Involvement in lateral medullary infarction patients with intracranial vertebral arterial lesions

Neurological sign/ symptoms	PICA +	PICA-
VG	3	4
NT	3	3
N/ E	5	3
DP	4	4
PVP	4	7
HS	1	8
PTH	5	8
LA	5	8
GA	7	8
MEN	8	6

## Discussion

According to the studies conducted by Fisher et al. regarding the lateral bulbar infarction, 14, 3% of the patients had PICA involvement, 38, 1% vertebral artery involvement (VA) and 26, 2% involvement of both arteries [**6**-**9**]. According to the study conducted above, 40% had a VA involvement (8 cases out of 20), 50 had an involvement of both arteries (10 out of 20) and 10% had a PICA involvement (2 cases out of 20). Regarding the neurological signs and symptoms, the results obtained in the patients studied were compared to those obtained by Jong S Kim, who performed one of the most extensive studies in the patients with lateral bulbar infarction (130 patients). In Kim’s study, the most frequent symptoms were the sensory disorders (96%) compared to the current study in which sensory disorders were obtained at a rate of 75%. Regarding the other signs and symptoms, Kim obtained the following results: gait ataxia 92%, Horner sign 88%, pharyngeal/ vocal cord paresis 88%, dysphagia 84%, vertigo 57%, nystagmus 56%, limb ataxia 55%, nausea/ emesis 52%. In the current study, gait ataxia was found in 90% of the patients, Horner sign in 60%, pharyngeal/ vocal cord paresis in 70%, dysphagia in 60%, vertigo in 45%, nystagmus in 45%, limb ataxia in 80%, nausea/ emesis in 35% [**10**]. The similar results between the two studies were obtained regarding the gait ataxia, the other symptoms and signs significantly differing in percentage (the above-mentioned data).

There were several limitations of the study. The small number of cases prevented the obtaining of certain generalizations. However, it should be mentioned that the lateral bulbar infarction is a quite rare pathology and that is why even the studies on small groups of patients are significant. Technical limitations: not all the hospitalized patients performed high-resolution contrast-enhanced three-dimensional imaging with spoiled gradient-recalled sequence (HR-CE 3D-SPGR) as it was initially desired and that is why only the patients who performed the 3D TOF common sequence with MIP were included in the study. 

**Acknowledgments**

Thanks and respect to Academician Professor Popa Constantin, MD, for the permanent and constant guidance. Without his involvement, this article would not have been possible. 

**Disclosures**

None
